# A Novel Missense Mutation in *TNNI3K* Causes Recessively Inherited Cardiac Conduction Disease in a Consanguineous Pakistani Family

**DOI:** 10.3390/genes12081282

**Published:** 2021-08-21

**Authors:** Shafaq Ramzan, Stephanie Tennstedt, Muhammad Tariq, Sheraz Khan, Hafiza Noor Ul Ayan, Aamir Ali, Matthias Munz, Holger Thiele, Asad Aslam Korejo, Abdul Razzaq Mughal, Syed Zahid Jamal, Peter Nürnberg, Shahid Mahmood Baig, Jeanette Erdmann, Ilyas Ahmad

**Affiliations:** 1 Institute for Cardiogenetics, University of Lübeck, 23562 Lübeck, Germany; shafaq.gagn@yahoo.com (S.R.); stephanie.tennstedt@uni-luebeck.de (S.T.); h.noorulayan@student.uni-luebeck.de (H.N.U.A.); matthias.munz@gmx.de (M.M.); jeanette.erdmann@uni-luebeck.de (J.E.); 2 National Institute for Biotechnology and Genetic Engineering (NIBGE-C), Institute of Engineering and Applied Sciences (PIEAS), Islamabad 44000, Pakistan; tariqpalai@gmail.com (M.T.); sherazkhanhu@gmail.com (S.K.); myaamirali@yahoo.com (A.A.); shahid_baig2002@yahoo.com (S.M.B.); 3 DZHK (German Research Centre for Cardiovascular Research) Partner Site Hamburg/Lübeck/Kiel, 23562 Lübeck, Germany; 4 University Heart Center Lübeck, 23562 Lübeck, Germany; 5 Cologne Center for Genomics (CCG), University of Cologne, Faculty of Medicine, University Hospital Cologne, 50931 Cologne, Germany; holger.thiele@uni-koeln.de (H.T.); nuernberg@uni-koeln.de (P.N.); 6 National Institute of Cardiovascular Disease, Karachi 75510, Pakistan; asadaslamkorejo@gmail.com (A.A.K.); drzahidjamal@yahoo.com (S.Z.J.); 7 Faisalabad Institute of Cardiology, Faisalabad 38000, Pakistan; a.razzaq.cs@gmail.com; 8 Center for Molecular Medicine Cologne (CMMC), University of Cologne, Faculty of Medicine, University Hospital Cologne, 50931 Cologne, Germany; 9 Department of Biological and Biomedical Sciences, Aga Khan University, Karachi 74000, Pakistan; 10 Pakistan Science Foundation (PSF), 1-Constitution Avenue, G-5/2, Islamabad 44000, Pakistan

**Keywords:** TNNI3K, missense mutation, molecular modeling simulation, cardiac conduction

## Abstract

Cardiac conduction disease (CCD), which causes altered electrical impulse propagation in the heart, is a life-threatening condition with high morbidity and mortality. It exhibits genetic and clinical heterogeneity with diverse pathomechanisms, but in most cases, it disrupts the synchronous activity of impulse-generating nodes and impulse-conduction underlying the normal heartbeat. In this study, we investigated a consanguineous Pakistani family comprised of four patients with CCD. We applied whole exome sequencing (WES) and co-segregation analysis, which identified a novel homozygous missense mutation (c.1531T>C;(p.Ser511Pro)) in the highly conserved kinase domain of the cardiac troponin I-interacting kinase (TNNI3K) encoding gene. The behaviors of mutant and native TNNI3K were compared by performing all-atom long-term molecular dynamics simulations, which revealed changes at the protein surface and in the hydrogen bond network. Furthermore, intra and intermolecular interaction analyses revealed that p.Ser511Pro causes structural variation in the ATP-binding pocket and the homodimer interface. These findings suggest p.Ser511Pro to be a pathogenic variant. Our study provides insights into how the variant perturbs the TNNI3K structure-function relationship, leading to a disease state. This is the first report of a recessive mutation in *TNNI3K* and the first mutation in this gene identified in the Pakistani population.

## 1. Introduction

Cardiac conduction disease (CCD) is a hypernym that encompasses numerous rare disorders in which the integrity of the heart conduction system is disrupted [1]. In CCD, anatomical or functional alterations in the conduction system lead to slowing or blocking of impulse induction, which can cause syncope or sudden death. Structural CCD with abnormal impulse propagation is most often a secondary impact of congenital heart disease or histological heart abnormalities [1,2]. Physiologically, CCD is primarily due to dysfunction of the sinoatrial node (SAN), atrioventricular node (AVN), His-bundle, the right or left bundle branch (RBB or LBB) Purkinje system, or the peripheral ventricular conduction system (PVCS) [3,4]. The molecular and cellular mechanisms underlying CCD are diverse; however, regardless of its cause, pacemaker therapy is a potential cure. Understanding of genetic contributions to cardiac electrophysiology has been advanced by familial clustering and GWAS studies, although the latter approach has had limited success in identification of genetic variants associated with phenotypic consequences [5,6].

Familial aggregation of conduction system diseases has been highly valuable in uncovering the molecular mechanisms underlying CCD and establishing phenotype-genotype correlations [4,6]. Most conduction diseases are inherited in an autosomal dominant manner; however, a few rare homozygous mutations have revealed recessive inheritance patterns [6,7]. Pedigree and large cohort studies have been used successfully to identify causative genes implicated in dysrhythmias and conduction disorders [6,7,8]. To date, the identified genes harboring rare variant encoding proteins with diverse functions include ion channels, circulating hormones, cardiac transcription factors, and structural proteins such as SCN5A (MIM 600163), SCN1B (MIM 600235), KCNK17 (MIM 607370), KCNQ1 (MIM 607542), NPPA (MIM 108780), CX40 (MIM 121013), TRPM4 (MIM 606936), NKX2.5 (MIM 1 600584), LMNA (MIM 150330), and TNNI3K (MIM 613932) [9,10,11,12,13,14,15,16,17].

The human cardiac troponin I-interacting kinase (*TNNI3K*) gene maps to chromosome 1p31.1, comprises 25 exons, and encodes a protein of 835 amino acids, with a molecular mass of 93 kDa [18]. TNNI3K is a dual-functional (serine/threonine and tyrosine) kinase that modulates its target proteins by transferring a phosphate group from ATP on to the hydroxyl group of Ser or Thr (during Ser/Thr kinase activity) or Tyr (during Tyr kinase activity). TNNI3K contains four domains: an N-terminal coiled-coil domain (amino acids (aa) 21–51); ten ankyrin (ANK) repeats in the amino terminus (aa 66–410); a central active kinase domain (aa 463–723); and a COOH-terminal serine-rich domain (aa 733–746) [18]. Furthermore, TNNI3K has numerous functional binding sites in its molecular structure, including those for ATP (Ser499), magnesium (site unknown), cardiac troponin I (cTnI) (site unknown), MEF2 (aa 147–157), TBX5 (aa 74–89), SRE (aa 82–96), M-CAT box (aa 120–135), and GATA4 (aa 160–169), indicating that it has many interacting partners [18,19]. Among these interacting proteins, cTnI (mutant forms of which can cause hypertrophic cardiomyopathy) directly interacts with TNNI3K [19] Furthermore, homology search and phylogenetic analysis indicate that TNNI3K belongs to a tyrosine kinase-like (TLK) group within the mixed lineage kinase (MLK) kinase kinase (MAPKKKS or MAP3K) family in the human kinome tree [20,21]. The regulation and activation of MAP3K kinases are poorly understood; however, several of them, including MEKK2 (MAP/ERK kinase kinase 2), dual leucine zipper-bearing kinase (DLK), apoptosis signal-regulating kinase 1 (ASK1), and integrin-linked kinase (ILK), form dimers or oligomers in response to upstream stimuli and these dimeric/oligomeric states are considered essential for downstream signal transduction [21,22]. TNNI3K can form homodimers or oligomers that lead to its self-phosphorylation and activation. The N-terminal ANK domain is necessary for autophosphorylation, while the C-terminal domain negatively regulates kinase activity [21].

TNNI3K is a cardiomyocyte-specific protein kinase with key roles in cardiac contractility, hypertrophic remodeling, and cardiac differentiation, and helps to repair cardiac injury by suppressing cTnI, which facilitates the maintenance of heartbeat rhythm and contractual force [23,24]. Mutations in *TNNI3K* have been linked to numerous cardiac phenotypes including cardiac conduction, atrial/junctional tachycardia, dilated cardiomyopathy, and heart failure [16,25,26,27]. In a mouse model, *Tnni3k* knock-out resulted in enhanced cardiomyocyte proliferation following injury, while overexpression of the molecule in zebrafish had different effects, including stimulation of cardiomyocyte polyploidy and impairment of cardiac regeneration [28]. To date, five single nucleotide variants have been reported in human *TNNI3K*. Among them, three missense, one splicing, and one nonsense mutation were reported in different patients from Germany, Canada, Salvador, and China [16,25,26,27]. Here, we report a novel recessive missense variant in *TNNI3K* (NM_015978.2: c.1531T>C) in a Pakistani family, which was not present in the Human Gene Mutation Database (HGMD) or any other publicly accessible databases.

## 2. Materials and Methods

### 2.1. Human Subjects, DNA Extraction, and Clinical Evaluation

**Figure 1:**
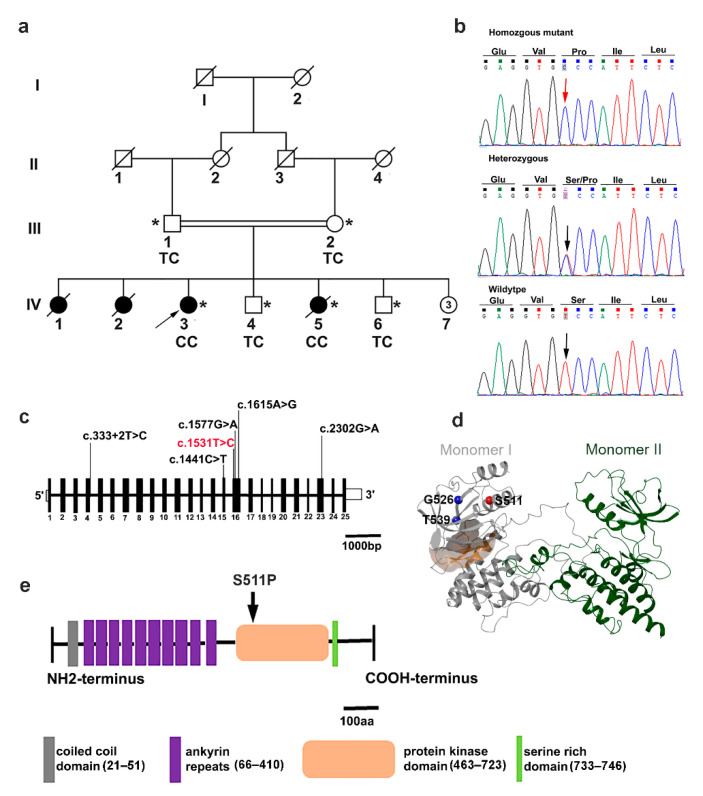
Pedigree, sequencing chromatogram, and location of the *TNNI3K* variant in the family with cardiac conduction disease (CCD). (**a**) Family pedigree. The open symbols represent unaffected individuals and the filled symbols represent affected individuals; symbols with a diagonal line represent deceased individuals; and the arrowhead designates the proband. An asterisk indicates family members from whom DNA was available. The genotypes of the *TNNI3K* mutation are indicated below each examined member: CC (homozygote); TC (heterozygote); number inside the circle denote the number of individuals. (**b**) Sequencing chromatograms. Vertical arrows indicate the mutation site. (**c**) Schematic of the human TNNI3K gene. The positions of coding exons (black) and UTRs (white) are indicated. Black arrows indicate previously reported pathogenic variants and the red arrow shows the novel variant c.1531T>C in exon 16. (**d**) Initial 3D structure of TNNI3K-WT (PDB-ID: 4YFI [29]) shown as a ribbon. Monomer I is shown in gray and monomer II in green. The Cα atoms of Ser511 (red), Gly526 (blue), and Thr539 (blue) of monomer I are shown in spheres relative to the ATP-binding pocket (orange surface) of monomer I. Gly526 and Thr539 are known TNNI3K missense variants. **(e)** TNNI3K protein domain structure. Coiled coil domain (gray); functional ankyrin repeat domains, ANK1–ANK10 (purple); kinase domain (orange), where the homozygous mutation p.Ser511Pro resides (arrowed); and a serine-rich domain (spring green).

A four-generation consanguineous family with conduction heart disease was recruited from Swat, Khyber Pukhtunkhwa, Pakistan (Figure 1a). Written informed consent was obtained from all adult individuals and the parents of patients. Blood samples from two affected and four unaffected individuals were obtained in ethylenediaminetetraacetic acid-containing BD vacutainers^®^ (BD-Plymouth, Plymouth, UK). DNA from blood samples was isolated using the Qiamp DNA FFPE Tissue kit (Qiagen, Hilden, Germany). Phenotypic assessment and diagnoses were conducted in two living symptomatic family members based on cardiological investigations and clinical data. Rhythm and conduction abnormalities were detected by a standard 24 h Holter electrocardiogram (ECG) and 12-lead surface ECG (speed 25 mm/s, 10 mm/mV). Echocardiography was performed to check for problems with the valves or chambers of the heart.

### 2.2. Whole Exome Capture and Sequencing

WES of two of the affected individuals (IV-3 and IV-5), their non-affected siblings (1V-4 and IV-6), and their parents (III-1 and III-2) (Figure 1a) were carried out at the Cologne Center for Genomics (CCG, University of Cologne, Cologne, Germany). A genomic DNA sample (1 µg) was sheared by fragmentation (Covaris, Woburn, MA, USA) and purified for library preparation using Agencourt AMPure XP beads (Beckman Coulter, Fullerton, CA, USA) following the manufacturer’s protocol. Enrichment was carried out using the Agilent SureSelect Human All Exon V6 r2 kit (Agilent Technologies, Santa Clara, CA, USA). Captured libraries were subjected to paired-end sequencing on an Illumina HiSeq 4000 system (Illumina, San Diego, CA, USA) using a paired end 2 × 75bp protocol. Base calling and demultiplexing were performed using the Illumina CASAVA pipeline and v1.7. Raw reads were mapped against the human reference genome hg38/GRCh38 (Genome Reference Consortium Human GRCh38) using the BWA alignment algorithm [30]. To reduce noise and false positive calls, PCR duplicates were removed using Picard, followed by local and base quality score recalibration using the Genome Analysis Toolkit (GATK, v3.6) [31]. Single nucleotide polymorphisms and short insertions/deletions (INDELs) were called using tools including GATK HaplotypeCaller (v3.6), SAMtools mpileup (v1.6), and Platypus (v0.8.1) [32,33,34]. Subsequently, base quality scores were recalibrated to correct sequencing errors and experimental artifacts using GATK v3.6 [31]. The ExomeDepth, Conifer, and XHMM algorithms were applied to detect copy number variants and ALLEGRO was used to identify regions with runs of homozygosity (ROH) [33,35,36,37]. Software tools developed at CCG were also employed, including the COMBINE module, which uses an INDEL normalization step to integrate INDEL calls in repetitive sequence environments, and the FUNC module for a functional interpretation of variations. Exome sequencing data were processed and analyzed using the Varbank V2.12 exome pipeline from CCG (https://varbank.ccg.uni-koeln.de/varbank2/ (accessed on 3 November 2018)). The following criteria were applied to filter rare and homozygous variants: coverage > 5 reads; quality score > 10; allele read frequency ≥ 75%; minor allele frequency (MAF) < 0.001 in gnomAD; in-house population allele frequency  <  0.001; strand bias estimated using Fisher’s Exact Test (FS) <  40; MQRankSum ≥ −5; QD > 5; MQ > 50; ReadPosRankSum ≥ −5; quorum level indel = 1; mapping quality >  50; quorum level SNV = 2; splice site score change ≤ 15%; and translation initiation site (+score change > 15%, −score change −15%) [38,39]. Analysis focused on single nucleotide, non-synonymous, missense, loss-of-function, and splice site variants, as well as short INDELs. The resulting gene lists were prioritized based on scores obtained from the dbNSFP/dbSCSNV v3.4 databases (filtering was based on normalized rank scores, which range from 0 = benign to 1 = pathogenic) and variants that were located in regions with ROH [35,40]. Additionally, data from various public databases (dbSNP; 1000 Genomes, dbVar) were used to determine the distribution of genetic variants in large populations and disease-specific databases (commercial HGMD professional database; ClinVar, OMIM) were screened to determine whether variants had any associated phenotypes. High priority variants were validated by Sanger sequencing.

### 2.3. Sanger Sequencing Analysis

Primer pairs were designed using Primer 3 software. PCR products were directly sequenced using a commercial service (Microsynth AG, Sankt Gallen, Switzerland). Segregation analysis of DNA from all available family members was performed by bidirectional Sanger sequencing to validate mutations and sequences were analyzed using DNASTAR Lasergene12 (DNASTAR Inc., Madison, WI, USA). Primer sequences are listed in Table S1.

### 2.4. Molecular Dynamic Simulations

For the molecular dynamic (MD) simulation, the X-ray structure of wild-type (WT) TNNI3K was retrieved from the Protein Data Bank (PDB; http://www.rcsb.org/; download on 15 August 2020; PDB-ID: 4YFI) and converted into its biological oligomerization state [29,41]. The mutant structure of TNNI3K (containing p.Ser511Pro) was built based on the WT X-ray structure, with an introduction of the single-point mutation. The WT and mutant structures were preprocessed using the Protein Preparation Wizard (SCHRÖDINGER^®^ 2020-3) [42,43]. Bond orders were assigned, all hydrogen atoms were added to each structure, and termini were capped. Incomplete side chains and loops were calculated and water molecules deleted. Heterogenetic states were generated at pH 7 ± 2. Hydrogen bonding networks were optimized by minimization of sampled hydrogens [44]. Each structure was minimized to a gradient of 0.05 using an OPLS3e force field. Finally, the minimized structures were subjected to solvent-explicit all-atom MD simulations using the GPU-accelerated SCHRÖDINGER^®^ DESMOND software [45,46]. Each structure was integrated in a minimized periodic simple point charged water model with an orthorhombic box size of 10.0 Å × 10.0 Å × 10.0 Å and 0.15 M NaCl ions. Each solvated system was minimized and relaxed using the default protocol of SCHRÖDINGER^®^ Desmond. MD simulations were carried out with periodic boundary conditions in the NPT ensemble using OPLS3e force field parameters. Temperature and pressure were maintained at 300 K and 1 atmospheric pressure, respectively, using Nose-Hoover temperature coupling and isotropic scaling. The operation was followed by running a 1 µs NPT production simulation for each structure, saved at the 1 ns interval. Three independent MD simulations were conducted for each case.

Next, the WT and mutated structures were compared based on root mean square deviation (RMSD) and root mean square fluctuation (RMSF). Residue-residue contacts between WT and mutant structures were monitored over the course of the last 200 ns of the simulation time using the trajectory_asl_monitor.py script supported by SCHRÖDINGER^®^. A contact was defined as a distance of < 5 Å between the centers of masses of defined contact residue pairs. For each residue contact pair, the fraction of the simulation time in which the residues were in contact was calculated as a mean of triplicate measurements. Interaction energy values between two monomers for the WT and mutant structures were computed using the Molecular Mechanics (MM)/Generalized Born Surface Area (GBSA) method implemented in SCHRÖDINGER^®^. The average binding free energy (ΔG_bind), based on MM/GBSA, was calculated using the SCHRÖDINGER^®^ Desmond command-line thermal_mmgbsa.py script. During the MM/GBSA calculation, the last 200 ns MD simulation trajectory (every 10th snapshot) was used as an input to compute the average binding free energy. All heatmaps and plots were generated using GraphPad Prism version 9.0.0 for Windows (GraphPad Software, San Diego, CA USA, www.graphpad.com). Figures were created using SCHRÖDINGER^®^ Meastro 2020-3 [47].

## 3. Results

### 3.1. Clinical Features

Early onset cardiac conduction disease (CCD) was clinically diagnosed in four members of a Pakistani family (Figure 1a). The proband (IV-3) was a 22-year-old female with a cardiac conduction defect and no underlying structural heart anomalies. A 24 h Holter recording revealed mild sinus bradycardia with an average heart rate of 55 beats per minute (bpm) at rest (range: 24–110) and sinus tachycardia of 160 bpm during exercise. Standard 12-lead ECG recorded at rest showed changes in the right bundle branch block and left anterior fascicular block (RBBB/LAFB) with a bifascicular block. In individual IV-5, the 24 h Holter monitor recorded bradycardia with an average heart rate of 56 bpm and tachycardia of 160 bpm; however, the echocardiogram revealed a restrictive perimembranous ventricular septal defect with bilateral shunt. This patient died during this study due to sudden cardiac arrest (SCA). In addition, two other siblings (IV 1 and IV 2) who had a history of CCD died due to SCA at 26 and 23 years-of-age, respectively. Both parents and healthy siblings were assessed for CCD but no relevant clinical features were detected. Clinical data are presented in Table 1.

### 3.2. Exome Sequencing Reveals a Pathogenic TNNI3K Variant

To uncover the underlying genetic cause of CCD in the family, we performed exome sequencing in all available individuals, including two affected individuals, their parents, and two healthy siblings (Figure 1a). We assumed an autosomal-recessive mode of inheritance because of the consanguinity of the healthy parents. After applying filtering criteria as described in Section 2.2, a total of 81 rare genetic variants, including 79 non-synonymous and two synonymous variants, were identified (Figure S1). The identified variants spanned 69 protein coding genes and seven non-coding genes. Among the variants, eight co-segregated with the phenotype in the pedigree (Table S2). Candidate variants were further screened by filtering within regions containing runs of homozygosity (ROH) (Table S3). Of the three resulting candidate variants, the *TTLL7* (NM_001350215.1) missense variant is predicted to lead a serine to alanine change in a moderately conserved amino acid, which lies outside of protein domains with known functions*; TTLL7* is expressed at high levels in the mammalian nervous system. The MET (NM_000245.3, NM_001324402.1) missense variant (rs545332056) changes an alanine to serine at position 319; this is a highly conserved amino acid but reported in ClinVar (CA4448049) as of uncertain significance. Interestingly, a homozygous missense mutation (Phe841Val) in MET was reported in a Pakistani family with hearing impairment; however, no such phenotype was detected in any affected individuals in the current study. The TNNI3K (NM_015978.2) serine residue at position 511 is highly conserved in TNNI3K orthologues; accordingly, p.Ser511Pro is classified as “damaging” based on SIFT and Polyphen2 scores (Figure S2, Table S3). It also had very high dbNSFP and phred-scaled CADD (v1.3) scores of 0.79 and 26.9, respectively, consistent with pathogenicity (Table S3). Moreover, mutations in *TNNI3K* have also previously been linked to the conduction heart disease spectrum (MIM 616117) (Table S4). Therefore, the variant in *TNNI3K* is the most likely causative candidate for the phenotype under investigation. The identified variant is an extremely rare homozygous variant that has not been reported in a heterozygous/homozygous state in publicly available exome/genome databases. Furthermore, it was absent from 110 exomes from Pakistani families with various heart diseases and in exome sequence data from 50,000 UK Biobank participants.

### 3.3. In Silico Molecular Dynamic Simulation

#### 3.3.1. p.Ser511Pro Influences TNNI3K Structural Fluctuation

**Figure 2:**
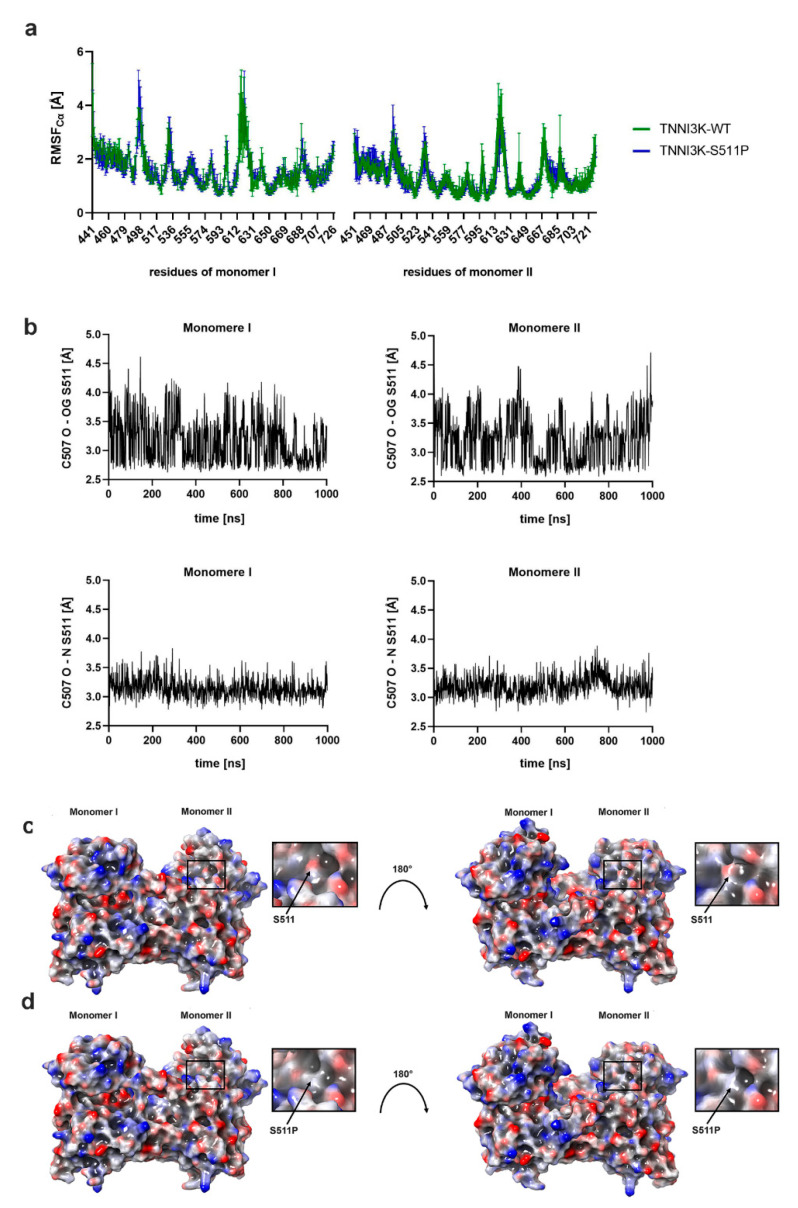
Root mean square fluctuation, hydrogen bond analysis, and electrostatic surface potential map. (**a**) Comparison of root mean square fluctuation values of TNNI3K-WT (green) and TNNI3K-S511P (blue) during three independent 1 µs MD simulations with error bars. Alignments and measurements were performed for the Cα carbon atoms. (**b**) Hydrogen bond analysis. Length of the hydrogen bonds between Cys507 and Ser511 in TNNI3K-WT as a mean value of three independent MD simulations (1 µs). (**c,d**) A comparison of the electrostatic surface potential of TNNI3K-WT (**c**) and TNNI3K-S511P (**d**), demonstrating that p.Ser511Pro leads to a more hydrophobic protein surface (“red white blue” color ramp with a minimum of −0.3 and a maximum of 0.3).

To understand the effect of p.Ser511Pro on the structural and dynamic properties of the protein, we conducted three independent 1 µs MD stimulations on the wild type and mutant structures. The X-ray structure of TNNI3K with the lowest resolution (PDB-ID: 4YFI [29]), containing amino acids Glu441 to Ile726 for monomer I (chain A) and amino acids Gly451 to Met721 for monomer II (chain B), was used for the analysis of the wild type model (TNNI3K-WT; Figure 1d). The mutant model (TNNI3K-S511P) was obtained by introducing a point mutation (p.Ser511Pro) into TNNI3K-WT. The resulting RMSD_Cα_ values of the MD simulations indicated that both models reached an equilibrium state within a simulation time of 1 µs (Figure S3a). The distinct conformational states sampled for both models showed no major differences (Figure S3b). Structural fluctuation of TNNI3K-P511S was comparable to that of TNNI3K-WT over the entire lengths of the models (average RMSF_Cα_: 1.0 Å (TNNI3K-WT) and 1.1 Å (TNNI3K-S511P); Figure 2a). The only pronounced differences were seen in the ATP-binding pocket of monomer I (Leu495-Leu496) and in the dimer interface of monomer II (Thr637, Met677, and His680-Ile682). All other regions exhibited changes smaller than the standard deviation or changes below 0.5 Å.

#### 3.3.2. p.Ser511Pro Affects the Local Hydrogen Bonding Network and Protein Surface Hydrophobicity of TNNI3K

Next, we investigated the local effect of p.Ser511Pro, which is located in helix 2 (Lys500-Asn517). Ser511 formed one hydrogen bond from its backbone nitrogen to the backbone oxygen of Cys507 and a second hydrogen bond from its side chain hydroxyl oxygen to the backbone oxygen of Cys507 throughout the simulation time (Figure 2b). Interestingly, p.Ser511Pro cannot form a hydrogen bond to Cys507 because of its specific structure. Furthermore, the protein surface of TNNI3K-S511P was more hydrophobic than that of TNNI3K-WT (Figure 2c,d).

#### 3.3.3. p.Ser511Pro Affects the Structure of Helix 2 and the ATP-Binding Pocket

**Figure 3:**
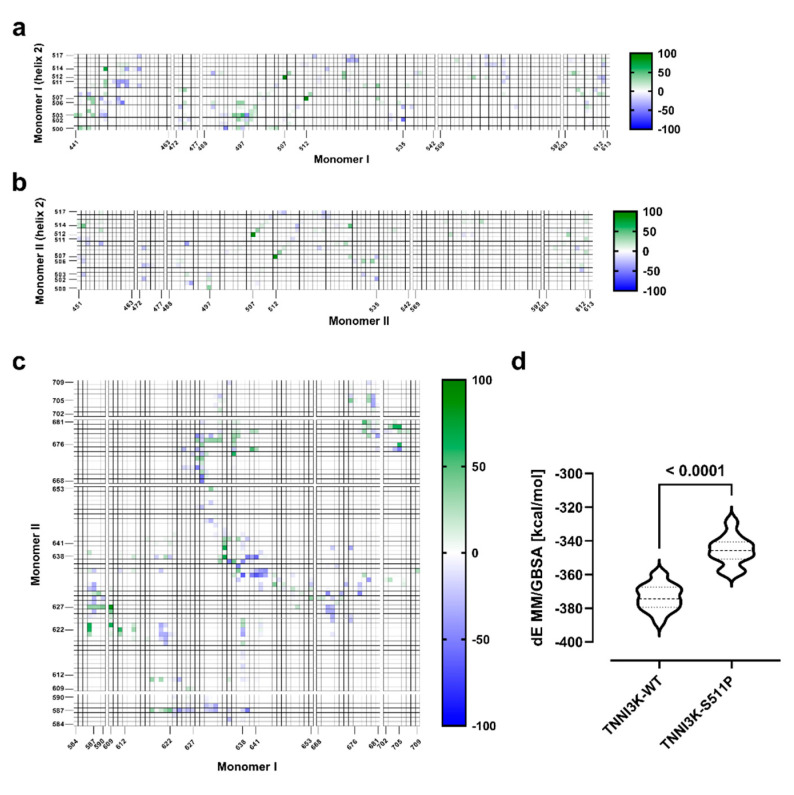
Average intra/inter residue-residue contact maps and MM/GBSA analysis. (**a**) Contact map of the residues of helix 2 in monomer I and the residues of the corresponding monomer, and (**b**) residues of helix 2 in monomer II and the residues of the corresponding monomer. Each point represents the mean of the average residue-residue contact difference between TNNI3K-WT and TNNI3K-S511P over the last 200 ns from the three independent MD simulations (spectrum range from green to white to blue, from 100% to −100% contact; green means more contacts in TNNI3K-WT and blue means more contacts in TNNI3K-S511P). (**c**) Contact map between residue-residue pairs in the dimer interface for TNNI3K-WT vs. TNNI3K-S511P. The contact spectrum ranging from green to white to blue, representing 100% to −100% contact, where green indicates more contacts in TNNI3K-WT and blue indicates more contacts in TNNI3K-S511P. Each point represents the mean difference in the residue-residue contact over the last 200 ns from the three independent MD simulations. (**d**) MM/GBSA analysis applied for dimerization of TNNI3K. Every 10th snapshot of the last 200 ns of the MD simulation was used. Each violin plot represents the mean from the three independent simulations (95% confidence interval).

To analyze the effects of p.Ser511Pro on each monomer structure, a comparative time-dependent intra-contact analysis was performed for TNNI3K-WT and TNNI3K-S511P between the amino acids of helix 2 and the amino acids of the corresponding monomer. As illustrated in Figure 3a,b, the largest contact difference of 97% (TNNI3K-WT: 98%; TNNI3K-S511P: 1%) in monomer I was found for the contact between Cys507 and Ile512, which are neighboring amino acids of S511 and S511P, respectively. For monomer II, the largest contact difference (91%) was also between Cys507 and Ile512 (TNNI3K-WT: 97%; TNNI3K-S511P: 6%). In monomer I, the distances between the Cα atoms of Ile512 and Cys507 were 6.02 ± 0.18 Å for TNNI3K-WT and 7.05 ± 0.40 Å for TNNI3K-S511P, respectively. Similar observations were made for monomer II (TNNI3K-WT: 6.03 ± 0.19 Å; TNNI3K-S511P: 6.99 ± 0.28 Å). These data imply that p.Ser511Pro causes minimal stretching of helix 2 in the mutant region (Cys507-Ile512) relative to TNNI3K-WT. Intriguingly, >±50% differences were further observed only for contacts between helix 2 amino acids, namely Asp502, Val503, Phe506 and Cys514, in monomer I and amino acids of the ATP-binding pocket (Phe448, Leu452, Tyr497, and Phe535; Figure 3a,b).

#### 3.3.4. p.Ser511Pro Creates a Less Favorable Binding Site at the Dimer Interface

In addition, amino acids at the dimer interface (Ile584-Asn590 and Glu609-Leu613; Figure S4) were located within a 10 Å radius around helix 2. Therefore, differences of ≤±30% were measured in intra contact analysis. The largest difference (−30%) was for the contact between Ile512 and Phe612 in monomer I (TNNI3K-WT: 70%; TNNI3K-S511P: 100%; Figure 3a,b). The Cα atom distance between Ile512 and Phe612 in monomer I was 10.29 ± 1.48 Å for TNNI3K-WT and 7.67 ± 0.68 Å for TNNI3K-S511P. Interestingly, Ile512 and Phe612 in monomer II showed a difference of only 2% (TNNI3K-WT: 100%, TNNI3K-S511P: 98%) with a Cα distance of 10.01 ± 0.62 Å for TNNI3K-WT and 10.17 ± 0.75 Å for TNNI3K-S511P. These findings indicate that for monomer I, Phe612, located in the dimer interface loop (Glu609-Leu613), is brought closer to helix 2 by the p.Ser511Pro mutation. Therefore, we hypothesized that p.Ser511Pro might indirectly influence the dimerization of TNNI3K. To assess this, we conducted a comparative time-dependent inter-contact analysis between the dimer interface amino acids (monomer I/II: Ile584-Ans590, Glu609-Leu653, Leu668-His681, and Trp702-Arg709; Figure S4) of TNNI3K-WT and TNNI3K-S511P in monomer I, an amino acid close to Phe612, and Asn627 in monomer II (TNNI3K-WT: 89.50%; TNNI3K-S511P: 5.67%). By contrast, Glu609 in monomer II and Asn627 in monomer I had contact values of 39.00% in TNNI3K-WT and 31.33% in TNNI3K-S511P, representing a difference of 7.67%. This is not surprising as Phe612 and Glu512 only showed strong differences in monomer I and not in monomer II. Similar results were observed for the contact between Cys705 and Asp676 (Cys705 monomer I −Asp676 monomer II: 59.33%; Asp676 monomer I −Cys705 monomer II: 5%). By contrast, the contact between Arg587 monomer I and Thr622 monomer II showed similar effects to those of their respective counterparts (Arg587 monomer I −Thr622 monomer II: 60.66%; Thr622 monomer I −Arg587 monomer II: 42.33%). Conversely, for Arg641 monomer I and Gln639 monomer II, more contacts were measured in TNNI3K-S511P than in TNNI3K-WT (Arg641 monomer I. −Gln639 monomer II: −58.66%; Gln639 monomer I −Arg641 monomer II: −10.33%). For all other 17 contacts, the counterpart went in the opposite direction. Together, these results indicate that the mutation affects the dimer-binding interface. To better understand the observed effects of the mutations on the dimer interface, we also employed the MM/GBSA method to calculate the binding energy between the two monomers; this is a simple post-processing technique in which the free energy of a state is calculated from the internal energy (MM) of the molecule and its interaction with an implicit representation of the solvent (GBSA). Although this method does not estimate the unfavorable entropy contribution to the binding and hence is less accurate than, for example, free energy perturbation, thermodynamic integration, or Bennett acceptance ratio, it avoids the enormous costs associated with other techniques and is consequently used often for large systems [48,49,50]. We considered the MM/GBSA values determined here as scores indicating predictive power in the ranking rather than representing quantitative agreement with experimentally observed affinities. The calculated MM/GBSA values (Figure 3d) indicate that the observed structural change leads to a less favorable total free binding energy and thereby to a less favorable bound state.

## 4. Discussion

In the present study, we identified a recessive CCD-associated sequence alteration, c.1531T>C;(p.Ser511Pro), in *TNNI3K* in a consanguineous Pakistani family. This variant affects an amino acid that is completely evolutionarily conserved (Figure S2) and which is predicted to be a causative mutation using *in silico* prediction tools. The missense mutation co-segregated with cardiac conduction disease in the family with complete penetrance (Figure 1a). It resides within the kinase domain of TNNI3K, which is highly conserved across species. This study aimed to gain insights into the effect of this mutation on the kinase domain and to assess its functional and structural consequences using long-term MD simulations.

The MD simulations provided detailed information about the structural fluctuation of the protein, the intra and intermolecular amino acid contacts, and the energetic properties of the mutant structure relative to the wild type. In the wild type model, Ser511 forms two hydrogen bonds with Cys507; one from its backbone nitrogen to the backbone oxygen of Cys507 and the other from its side-chain hydroxyl oxygen to the backbone nitrogen of Cys507. Pro511 cannot mimic the hydrogen bond network of Ser511 due to its unique structural property. This conformational change introduced by the mutation stretches helix 2 into the region containing the mutation (p.Cys507-Ile512) in both monomers.

Changes in both structural fluctuation (Figure 2a) and amino acid contacts (Figure 3a,b) were also observed in the monomer I ATP-binding pocket adjacent to helix 2. As amino acid orientation and interactions in the ATP-binding pocket are critical for the activity of the complex [51], these findings suggest that the mutation alters the ATPase activity of TNNI3K and therefore causes a reduction in its catalytic phosphorylation activity. Interestingly, our hypothesis is in line with the findings of Podliesna et al. [26] who observed a reduction or loss of autophosphorylation activity in all previously reported human TNNI3K kinase domain mutants (p.Gly526Asp and p.Thr539Ala). They postulated that affected individuals would exhibit reduced TNNI3K protein levels and kinase activity, eventually leading to a cardiac excitation conduction defect [26].

Moreover, using MD, we observed that the mutation in monomer I brings Phe612, located within a loop (Ala605 to Glu634) in the dimer interface, closer to helix 2. Further intercontact analysis confirmed changes in the region of the dimer interface. Together with the results of MM/GBSA-based analysis, we hypothesize that the change in the amino acid interaction pattern at the dimer interface results in a more energetically unfavorable dimer arrangement in TNNI3K-S511P. Further analyses using a heterozygous dimer will be required to obtain a deeper understanding of the one-sided effects of the mutation in the monomer I.

The mutation-induced change in helix 2 also influenced the hydrophobicity of the TNNI3K protein surface. It is known that the surfaces of proteins in an aqueous environment are predominantly composed of hydrophilic amino acids [52]. Therefore, the substitution of the polar serine to a non-polar proline led to an increase in the hydrophobicity of the protein surface (Figure 2c,d). Changes in surface hydrophobicity are closely associated with protein misfolding and aggregation, which are pathological hallmarks of many human diseases such as Alzheimer’s disease, Parkinson’s disease, and heart diseases [16,53]. Interestingly, previous studies have reported that a heterozygous *TNNI3K* missense mutation leading to a Gly526Asp substitution causes the formation of insoluble protein aggregates in *in vitro* analysis and a hydrophobic spot in the kinase domain surface in *in silico* protein surface analysis. Additionally, a dominantly negative mutation in TNNI3K (Gly526Asp) has been shown to deplete TNNI3K in ventricular cardiomyocytes [16]. Here, we show that a bi-allelic p.Ser511Pro mutation resulted in a recessive conduction defect in a Pakistani family.

Hitherto, disease-causing *TNNI3K* variants have been reported to be due to heterozygous mutations (Table S4). Symptoms manifest with age, with disease onset in CCD patients ranging from 18 to 84 years [16,25,26,27]. Interestingly, in the current study, the parents, who were obligate carriers, and heterozygous siblings were all asymptomatic; no subclinical evidence of cardiac conduction system dysfunction was observed. However, in homozygous patients, disease symptoms appeared at a very early age and patients had a shorter life expectancy than those in previous reports. Two patients (IV-I and IV-2) had already died at 26 and 23 years-of-age, respectively. A plausible explanation for these results may be autozygosity, which increases the homozygous occurrence of alleles associated with a dominant inheritance pattern. The genetic background and/or modifier loci could also have a role in increasing the severity of the disease [54,55,56].

To date, no studies have been conducted in the Pakistani population to investigate the genetics underlying the cardiac system, except for a report of a homozygous mutation in *CACNA1D*, which causes deafness with bradycardia syndrome [57]. Therefore, to the best of our knowledge, this is the first reported recessive *TNNI3K* mutation, as well as the first *TNNI3K* mutation of all detected in the Pakistani population.

Clinical implications: First, it is important to understand the underlying pathomechanisms of a disease; this is mainly facilitated by the identification of pathogenic mutations, especially in families, with a genetic background that is not frequently studied. Regardless, knowledge of a pathogenic mutation in a family helps in the early diagnosis of asymptomatic family members.

## 5. Conclusions

In summary, the p.Ser511Pro mutation identified in TNNI3K leads to a different hydrogen bond network in helix 2, which affects the contacts between amino acids of helix 2 and those of the ATP-binding pocket. It also affects the dimer interface, resulting in an unfavorable binding energy. As the p.Ser511Pro substitution results in greater hydrophobicity and loss of interaction with the ATP-binding pocket, Ser511 clearly plays an important role in orienting the TNNI3K dimer; however, these new findings require functional validation. Overall, our study contributes to the *TNNI3K* mutation spectrum and will inform genetic counseling for CCD in Pakistani families.

## Figures and Tables

**Table 1:** Clinical Details of the Family with Cardiac Conduction Disease (CCD).

Subject ID	Sex	Age (y)	Age at Diagnosis (y)	HR b.p.m.	Arrhy-thmia	Pacemaker Insertion	24 h ECG Holter	Echocardio-graphy	PR Interval	QRs Interval	HF	Other Associated Phenotypes	Health Status	*TNNI3K* Genotype
III-1	M	60	-	67	N	N	NAD	NAD	No AV block, 130 ms	Normal duration	N	-	U	TC
III-2	F	50	-	71	N	N	NAD	NAD	No AV block, 140 ms	PRWP	N	-	U	TC
IV-1	F	26	16	-	-	N	-	-	-	-	SCA	-	A	-
IV-2	F	23	14	-	-	N	-	-	-	-	SCA	-	A	-
IV-3	F	22	13	80	N	N	SB (55 beats/min) Tach (160 beats/min)	NAD	No AV block, 130 ms	RBBB, LAFB, PRWP, and BFB	N	Body posture defect	A	CC
IV-4	M	17	-	-	N	N	NAD	-	No AV block, 160 ms	LVH	N	-	U	TC
IV-5	F	15	12	80	N	N	SB (56 beats/min) Tach (160 beats/min)	VSD	-	-	SCA	-	A	CC
IV-6	M	10	-	77	N	N	NAD	NAD	No AV block, 140 ms	LAD, LAFB	N	-	U	TC

Note: ID indicates identification; age at most recent evaluation in years (y); HR, sinus heart rate; b.p.m, beats per minute; NAD, no abnormality detected; hyphen, no information, not present; SB, sinus bradycardia; Tach, Tachycardia; RBBB, right bundle branch block; PRWP, poor R wave progression; BFB, bifascicular block; LVH, left ventricular hypertrophy; LAD, left axis deviation; LAFB, left anterior fascicular block; HF, heart failure; SCA, sudden cardiac arrest; N, no; A, affected; U, unaffected; CC, homozygous mutant genotype; and TC, heterozygous genotype.

## Data Availability

All the data mentioned in this paper are available in the article and supplementary materials.

## Supplementary Materials

The following are available online at https://www.mdpi.com/article/10.3390/genes12081282/s1: Figure S1: Filtering and prioritizing homozygous genetic variants; Figure S2: Conservation of the TNNI3K-p.Ser511Pro mutation; Figure S3: Structural fluctuation of TNNI3K-WT (green) and TNNI3K-S511P (blue) during three independent 1 µs MD simulations; Figure S4: Dimer interface region of TNNI3K-WT (PDB-IDc: 4YF); Table S1: Primer sequence related to Sanger validation. Table S2: List of homozygous rare variants segregated with the observed phenotype; Table S3: List of primary homozygous candidate variants in runs of homozygosity (ROH) regions; and Table S4: The geographical distribution of *TNNI3K* pathogenic variants.
