# Supplementary material for: A Novel Missense Mutation in TNNI3K Causes Recessively Inherited Cardiac Conduction Disease in a Consanguineous Pakistani Family
Source: Genes (Basel). 2021 Aug 21;12(8):1282. doi: 10.3390/genes12081282 (PMC8395014; doi:10.3390/genes12081282)
Supplement: Supplementary file 1 [file genes-12-01282-s001.zip › genes-1311391-supplementary.pdf]

Supplementary Materials: Supporting information consists of four figures and four tables.

Table S1. Primer sequence, related to Sanger validation.

Name	Forward sequence	Reverse sequence
TNNI3K-Ex16	ATCCAGGTGGAATTGTGACC	AGGTGAAGGCTCATCCAATG

Exome Sequencing

Variant filtering was performed with the VARBANK graphical user interface (https://varbank.ccg.uni-koeln.de). Applying the standard filter criteria for rare and homozygous variant as discussed in the material and method section of this manuscript, 81 different variants (15 CNVs, 47 SNVs and 19 indels) were filtered at the first step.. Next filtration was performed based on complete penetrance; variants that were heterozygous in both parents and homozygous in affected individuals. To further narrow the search for disease causing variants, coding variants and canonical splice site variants (i.e., splice site donor and splice site acceptor variants) were retained and the rest were disregarded. In the last step, disease causative variant was selected based on OMIM database, ClinVar record, dbNSFP score, CADD score, Polyphen2 score, SIFT score, and literature research.

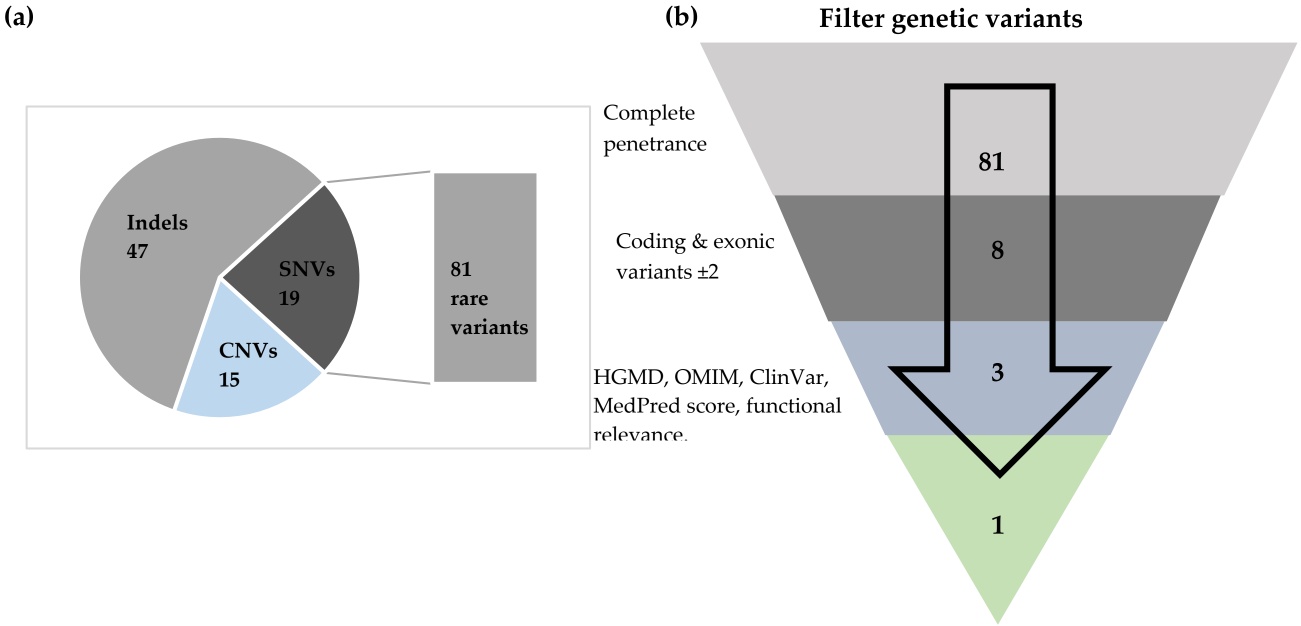

Figure S1. (a) Filtering and prioritizing homozygous genetic variants: **(a)** Overview of the different rare homozygous variants after assuming autosomal recessive inheritance using web interface of Varbank 2. **(b)** Filtering steps to find disease causing variant.

Table S2. List of homozygous rare variants segregated with the observed phenotype.

Chr	Start	End	Biotype	Type	EnsGene	ENsRNA	RefSeq	Gene	Consequ-ences	cDNA position c.	Protein position p.	Gnom AD Freq	dbSNP annotation	Med-Pred
1	74369449	74369449	antisense_RNA	SNV	ENSG00000237324	ENST00000415549	na	na	intragenic	n.108-330A>G	na	na	na	na
1	74369449	74369449	protein_coding	SNV	ENSG00000259030	ENST00000370899	NM_015978.2	*TNNI3K*	Missense	c.1988A>T	p. Asp663Val	na	na	0.79
1	83890325	83890325	protein_coding	SNV	ENSG00000137941	ENST00000260505	NM_024686.4,	*TTLL7*	Missense	c.2365T>G	p. Ser789Ala	0.0000994	rs778124501	0.55
1	92004622	92004622	protein_coding	SNV	ENSG00000137948	ENST00000402388.1	NM_001726.4	*BRDT*	Splice	c.2594+3C>T	na	na	na	na
9	92611269	92611554	protein_coding	CNV	ENSG00000188312	ENST00000375579	NM_001286971.1	*CENPP*	LOF	na	na	na	na	na
7	116700039	116700039	protein_coding	SNV	ENSG00000105976	ENST00000318493.11	NM_001127500.2	*MET*	Missense	c.955G>T	p. Ala319Ser	0.0000650	rs545332056	0.73
7	120994103	120994103	protein_coding	Intrag-enic	ENSG00000106034	ENST00000310396	NM_024913.4	*CPED1*	Intragenic	c.249+4233G>C	na	na	na	na
7	130385374	130385374	protein_coding	SNV	ENSG00000091704	ENST00000011292	NM_001868.3	*CPA1*	splice	c.987+29G>A	na	0.000208	rs534148807	na

These variants are segregating with the phenotype in the family. Chr, chromosome; start and end are the variant genomic position; Biotype, transcript biotype; EnsGene, ENSEMBL gene id; EnsRNA, ENSEMBL transcript id; RefSeq, RefSeq id provided by NCBI; na, not available; SNV, single nucleotide variant; CNV, copy number variation; intragenic, an intragenic variation without functional annotation; LOF, loss of function. MedPred scores were taken from the dbNSFP/dbSCSNV v3.4 databases. Filtering is based on the normalized rank scores, which range from 0=benign to 1=pathogenic.

Table S3. List of primary homozygous candidate variants in runs of homozygosity (ROH) regions.

Chr: region (build GRCh38/hg38)	Gene	RefSeq	Mut cDNA (Mut Prot)	Consanguin-ity/Zygosity	gnomAD Freq	dbSNP annotation	Med Pred	CADD PHRED V1.3	GERP++ RS	Poly-phene2	SIFT	ACMG interpretation	RVIS_ percentile	disease association (OMIM number, mode of inheritance)
1: 74369449	*TNNI3K*	NM_015978.2	c.1531T>C (p.Ser511Pro)	homozygous	na	na	0.79	26.8	5.27	1.0	0.009	Pathogenic (PS4, PM2, PP3, PPI, PP4)	29.5	Cardiac conduction disease with or without dilated cardiomyopathy (MIM 616117, AD)
7:116700039	*MET*	NM_000245.4	c.955G>T (p.Ala319Ser)	homzygous	0.0000650	rs545332056	0.73	25.9	6.04	1.0	0.0	VUS (BP5,BS2)	23.7	i)Deafness, autosomal recessive 97 (MIM 616705, AR)<br>ii) Osteofibrous dysplasia, susceptibility to (MIM 607278, AD)<br>iii) Hepatocellular carcinoma, childhood type, somatic (MIM 114550)<br>iv) Renal cell carcinoma, papillary, 1, familial and somatic (MIM 605074)
1: 83890325	*TTLL7*	NM_024686.4	c.2365T>G (p.Ser789Ala)	homzygous	0.0000994	rs778124501	0.55	23.8	5.12	0.8	0.007	Likely benign (BS2)	13.5	No association [1]

These filtered variants are located within the regions of homozygosity which are derived from exome sequence data. MedPred, CADD, GERP++_RS scores, Polyphen2, SIFT show the *in-silico* pathogenicity prediction of the variant. Variants were graded using the American College of Medical Genetics and Genomics (ACMG) classification system [2]. Abbreviation used; AR, autosomal recessive; AD, autosomal dominant; VUS, variant of uncertain significance; ACMG, American College of Medical Genetics and Genomics. The variant nomenclature was verified according to the Human Genome Variation Society (HGVS) standards using tool 'www.mutalyzer.nl.

Ser511Pro

```
Homo sapiens        481 RCRNKIVAIKRYRANTYCSKSDVDMFCREVSILCQLNHPCVIQFVGACLNDPSQFAIVTQ 540
Pan troglodytes     481 RCRNKIVAIKRYRANTYCSKSDVDMFCREVSILCQLNHPCVIQFVGACLNDPSQFAIVTQ 540
Macaca mulatta      481 RCRNKIVAIKRYRANTYCSKSDVDMFCREVSILCQLNHPCVIQFVGACLNDPSQFAIVTQ 540
Bos tauruss         481 RCRNKIVAIKRYRANTYCSKSDVDMFCREVSILCRLNHPCIIQFVGACLNDPSQFAIVTQ 540
Mus musculus        481 RCRNKIVAIKRYRANTYCSKSDVDMFCREVSILCQLNHPCVVQFVGACLDDPSQFAIVTQ 540
Ratus norvegicus    481 RCRNKIVAIKRYRANTYCSKSDVDMFCREVSILCQLNHPCVVQFVGACLDDPSQFAIVTQ 540
Canus lupus familia481 RCRNKIVAIKRYRANTYCSKSDVDMFCREVSILCRLNHPCIIQFVGACLNDPSQFAIVTQ 540
Gallus gallus       481 RCRNKIVAIKRYRANTYCSKSDVDMFCREVSILCRLNHPCVIQFVGACLDDPSQFAIVTQ 540
Xenopus tropicallis481 RCRNKIVAIKRYRANTYCSKSDVDMFCREVSILCRLNHPCVIQFVGACLDDPSQFAIVTQ 540
Danio rerio         481 KCRNKIVAIKRYRPNTYCSKSDTDMFCREVSILCRLNHPCVIQFVGACLDDPSQFAIVTQ 540
                        :************ ********.***********:*****::*******:**********
```

Figure S2: Conservation of TNNI3K-Ser511Pro mutation. Conservation analysis of the amino acid sequences between different species. The analysis was conducted using clustal omega. The mutation site and its neighboring sequences were well conserved among the different species. * Indicates identical amino acids indicates nonidentical residues. Numbers represent amino acid position.

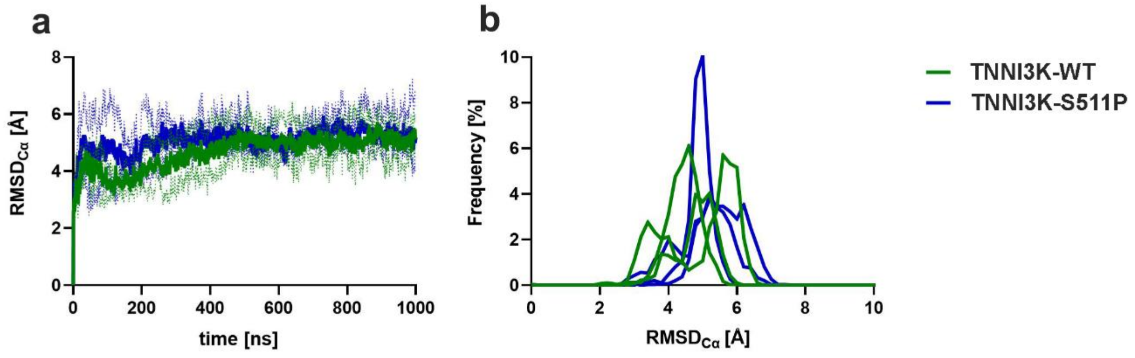

Figure S3: Structural fluctuation of TNN3K-WT (green) and TNN3K-Ser511Pro (blue) during three independent 1 μs MD simulations. Alignments and measurements were performed for the Cα carbon atoms. **(a)** Root mean square deviation (RMSD) plot, shown with respect to the initial minimized structures as mean values with error bars indicated by dots. **(b)** Distribution of RMSD.

Table S4. The geographical distribution of *TNNI3K* pathogenic variants.

AD, autosomal dominant; AR, autosomal recessive; rs, a reference SNP ID number; hypen, no information

No	*TNNI3K* variant	rsID	Type of mutation	Mode of inheritance	Family origin	Broad clinical phenotypes	Reference
1	c.1577G>A, p.Gly526Asp	rs606231469	Missense	AD	German	Conduction system disease, atrial tachyarrhythmia, dilated cardiomyopathy	Theis et al., (2014) [3]
2	c.1615A>G, p.Thr539Ala	rs1163083033	Missense	AD	Canada	Conduction system disease, congenital junctional ectopic tachycardia	Xi et al., (2015) [4]

3	c.333+2T>C, p.?	-	Splicing	AD	China	Conduction system disease, dilated cardiomyopathy	Fan et al., (2018) [5]
4	c.2302G>A, p.Gly768Leu	rs202238194	Missense	AD	Caucasian, Salvador	Supraventricular tachycardia, cardiomyopathy, conduction disease	Podliesna et al., (2019) [6]
5	c.1441C>T, p. Arg481*	rs757261707	Non-sense	AD	China	Cardiac conduction disease	Liu et al., 2020 [7]
6	c.1531T>C, p.Ser511Pro	-	Missense	AR	Pakistan	Cardiac conduction disease	This study

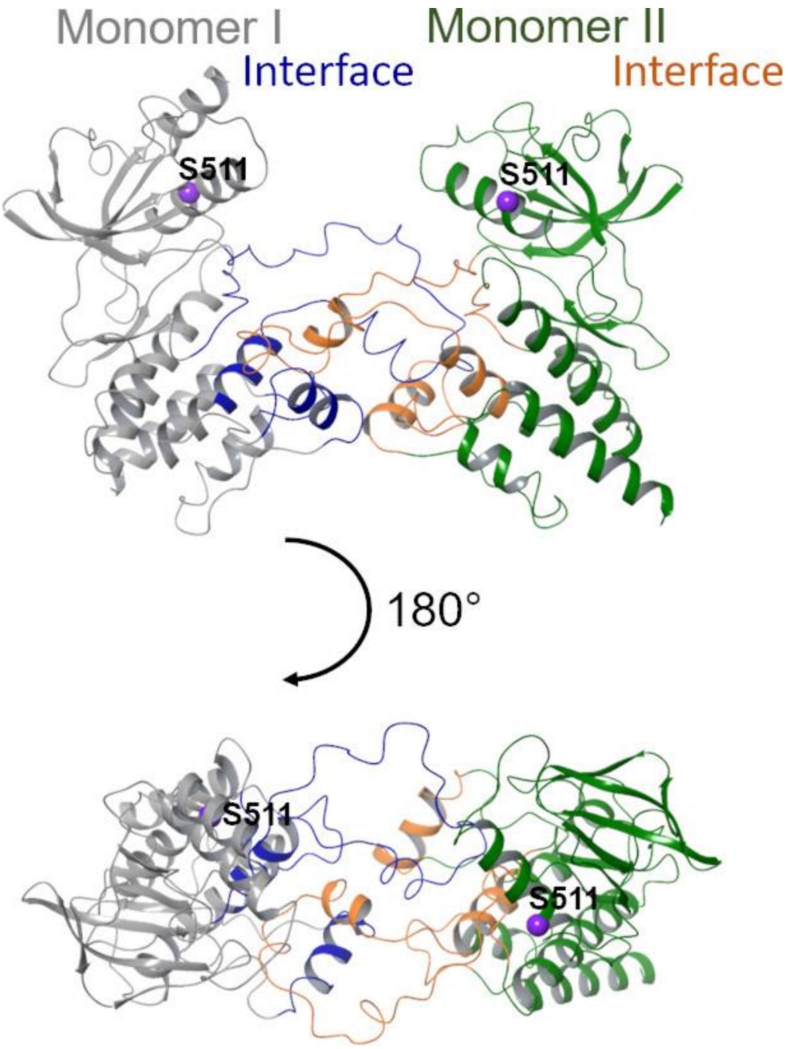

Figure S4: Dimer interface region of TNNI3K-WT (PDB-ID: 4YF3 [8]). Monomer I is represented as a gray ribbon with the amino acids of the dimer interface in blue and monomer II as a green ribbon with the amino acids of the dimer interface in orange. The Cα.atoms of Ser511 in monomers I and II are shown as violet spheres.

The URLs for data presented herein are as follows

- KiNET, http://www.kinexus.ca/kinet
- Mutalyzer, http://www. mutalyzer.nl/
- ENSEMBL, http://www.ensembl.org
- Online Mendelian Inheritance in Man (OMIM), www.omim.org
- Primer3, v.0.4.0, http://frodo.wi.mit.edu/primer3/input.htm
- SIFT (Sorting Intolerant from Tolerant), http://sift.jcvi.org/
- Picard, http://broadinstitute.github.io/picard/
- 1000 Genomes, http://browser.1000genomes.org
- dbSNP, http://www.ncbi.nlm.nih.gov/snp
- dbVar, http://www.ncbi.nlm.nih.gov/dbvar
- ClinVar, http://www.ncbi.nlm.nih.gov/clinvar
- OMIM, http://www.omim.org
- Human Gene Mutation Database, http://www.hgmd.org
- Varbank Variant Analysis Platform, https://varbank.ccg.uni-koeln.de/varbank2/

Supplementary References

1. Ikegami, K.; Mukai, M.; Tsuchida, J.; Heier, R.L.; Macgregor, G.R.; Setou, M. TTLL7 is a mammalian beta-tubulin polyglutamylase required for growth of MAP2-positive neurites. *J Biol Chem* **2006**, *281*, 30707-30716, doi:10.1074/jbc.M603984200.
2. Richards, S.; Aziz, N.; Bale, S.; Bick, D.; Das, S.; Gastier-Foster, J.; Grody, W.W.; Hegde, M.; Lyon, E.; Spector, E.; et al. Standards and guidelines for the interpretation of sequence variants: a joint consensus recommendation of the American College of Medical Genetics and Genomics and the Association for Molecular Pathology. *Genet Med* **2015**, *17*, 405-423, doi:10.1038/gim.2015.30.
3. Theis, J.L.; Zimmermann, M.T.; Larsen, B.T.; Rybakova, I.N.; Long, P.A.; Evans, J.M.; Middha, S.; de Andrade, M.; Moss, R.L.; Wieben, E.D.; et al. TNNI3K mutation in familial syndrome of conduction system disease, atrial tachyarrhythmia and dilated cardiomyopathy. *Hum Mol Genet* **2014**, *23*, 5793-5804, doi:10.1093/hmg/ddu297.
4. Xi, Y.; Honeywell, C.; Zhang, D.; Schwartzentruber, J.; Beaulieu, C.L.; Tetreault, M.; Hartley, T.; Marton, J.; Vidal, S.M.; Majewski, J.; et al. Whole exome sequencing identifies the TNNI3K gene as a cause of familial conduction system disease and congenital junctional ectopic tachycardia. *Int J Cardiol* **2015**, *185*, 114-116, doi:10.1016/j.ijcard.2015.03.130.

5. Fan, L.-L.; Huang, H.; Jin, J.-Y.; Li, J.-J.; Chen, Y.-Q.; Zhao, S.-P.; Xiang, R. Whole exome sequencing identifies a novel mutation (c. 333+ 2T> C) of TNNI3K in a Chinese family with dilated cardiomyopathy and cardiac conduction disease. *Gene* **2018**, *648*, 63-67, doi:10.1016/j.gene.2018.01.055.
6. Podliesna, S.; Delanne, J.; Miller, L.; Tester, D.J.; Uzunyan, M.; Yano, S.; Klerk, M.; Cannon, B.C.; Khongphatthanayothin, A.; Laurent, G.; et al. Supraventricular tachycardias, conduction disease, and cardiomyopathy in 3 families with the same rare variant in TNNI3K (p.Glu768Lys). *Heart Rhythm* **2019**, *16*, 98-105, doi:10.1016/j.hrthm.2018.07.015.
7. Liu, J.; Liu, D.; Li, M.; Wu, K.; Liu, N.; Zhao, C.; Shi, X.; Liu, Q. Identification of a nonsense mutation in TNNI3K associated with cardiac conduction disease. *J Clin Lab Anal* **2020**, *34*, e23418, doi:10.1002/jcla.23418
8. Lawhorn, B.G.; Philp, J.; Zhao, Y.; Louer, C.; Hammond, M.; Cheung, M.; Fries, H.; Graves, A.P.; Shewchuk, L.; Wang, L. Identification of purines and 7-deazapurines as potent and selective type I inhibitors of troponin I-interacting kinase (TNNI3K). *J Med Chem* **2015**, *58*, 7431-7448, doi:10.1021/acs.jmedchem.5b00931.
